# Supplementary figures and images for: Identification of potential gene signatures associated with osteosarcoma by integrated bioinformatics analysis
Source: PeerJ. 2021 May 27;9:e11496. doi: 10.7717/peerj.11496 (PMC8164836; doi:10.7717/peerj.11496)

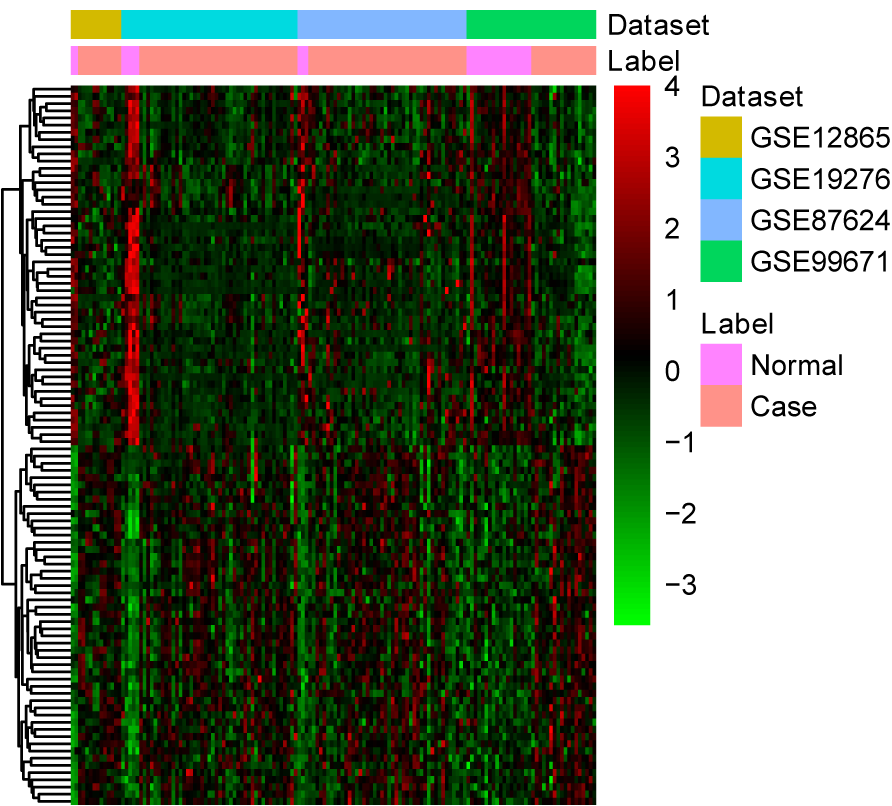

Supplement: Supplemental Information 1 [file peerj-09-11496-s001.tif]
