# Supplementary material for: Identification of potential gene signatures associated with osteosarcoma by integrated bioinformatics analysis
Source: PeerJ. 2021 May 27;9:e11496. doi: 10.7717/peerj.11496 (PMC8164836; doi:10.7717/peerj.11496)
Supplement: Supplemental Information 2 [file peerj-09-11496-s002.docx]

**Table S1 The information from selected GEO datasets in this study.**

| **GEO ID** | **Author, year** | **Platform** | **Country** | **Samples (T:N)** | **Common mRNAs** |
| --- | --- | --- | --- | --- | --- |
| GSE12865 | Sadikovic *et al*, 2009 | GPL6244 | Canada | 12:2 | 14981 |
| GSE19276 | Endo-Munoz *et al*, 2010 | GPL6848 | Australia | 44:5 |  |
| GSE87624 | Temiz *et al*, 2018 | GPL11154 | USA | 44:3 |  |
| GSE99671 | Kõks S *et al*, 2017 | GPL20148 | Estonia | 18:18 |  |
| GSE9508 | Endo-Munoz *et al*, 2010 | GPL6076 | Australia | 34:5 | / |

GSE9508 dataset contained over 50% missing data and was removed from this analysis.

*GEO* Gene Expression Omnibus; *N* normal controls; *P* patients with osteosarcoma.
