# Supplementary material for: Identification of potential gene signatures associated with osteosarcoma by integrated bioinformatics analysis
Source: PeerJ. 2021 May 27;9:e11496. doi: 10.7717/peerj.11496 (PMC8164836; doi:10.7717/peerj.11496)
Supplement: Supplemental Information 3 [file peerj-09-11496-s003.docx]

**Table S2 The differential expression of seven genes in validation dataset.**

| **Gene Symbol** | ***P* value** | **FDR** | **Regulation** |
| --- | --- | --- | --- |
| CAMP | 0.158 | 0.285 | Down |
| CXCL12 | 0.008 | 0.076 | Down |
| CYP4F3 | 0.072 | 0.144 | Down |
| LTF | 0.570 | 0.706 | Down |
| METTL7A | 0.047 | 0.115 | Down |
| NETO2 | 0.044 | 0.115 | Up |
| TCN1 | 0.036 | 0.115 | Down |

*FDR* False discovery rate
