# Supplementary material for: Identification of potential gene signatures associated with osteosarcoma by integrated bioinformatics analysis
Source: PeerJ. 2021 May 27;9:e11496. doi: 10.7717/peerj.11496 (PMC8164836; doi:10.7717/peerj.11496)
Supplement: Supplemental Information 4 [file peerj-09-11496-s004.docx]

**Table S3 The differential expression of seven genes in GSE126209 dataset.**

| **Gene Symbol** | ***P* value** | **FDR** | **Regulation** |
| --- | --- | --- | --- |
| CAMP | 0.340 | 0.340 | Down |
| CXCL12 | 0.011 | 0.039 | Down |
| CYP4F3 | 0.163 | 0.231 | Up |
| LTF | 0.332 | 0.340 | Up |
| METTL7A | 0.037 | 0.086 | Down |
| NETO2 | 0.002 | 0.020 | Up |
| TCN1 | 0.054 | 0.095 | Down |

*FDR* False discovery rate
